# Immunohistochemical analysis based Ep-ICD subcellular localization index (ESLI) is a novel marker for metastatic papillary thyroid microcarcinoma

**DOI:** 10.1186/1471-2407-12-523

**Published:** 2012-11-15

**Authors:** Tada Kunavisarut, Ipshita Kak, Christina MacMillan, Ranju Ralhan, Paul G Walfish

**Affiliations:** 1 Alex and Simona Shnaider Laboratory in Molecular Oncology, Department of Pathology & Laboratory Medicine, Samuel Lunenfeld Research Institute, Mount Sinai Hospital, 60 Murray Street, Suite L6-304, Toronto, ON, M5T 3L9, Canada; 2 Department of Pathology & Laboratory Medicine, Mount Sinai Hospital, Joseph & Wolf Lebovic Health Complex, 600 University Avenue, Room 6-423, Toronto, ON, M5G 1X5, Canada; 3 Joseph and Mildred Sonshine Family Centre for Head and Neck Diseases, Department of Otolaryngology-Head and Neck Surgery, Mount Sinai Hospital, 600 University Avenue, Toronto, ON, M5G 1X5, Canada; 4 Department of Medicine, Endocrine Division of Mount Sinai Hospital and University of Toronto Medical School, Toronto, ON, M5G 1X5, Canada; 5 Department of Otolaryngology-Head and Neck Surgery, University of Toronto, Toronto, ON, M5G 2N2, Canada; 6 Department of Medicine, Division of Endocrinology and Metabolism, Siriraj Hospital, Mahidol University, Bangkok, 10700, Thailand; 7 Joseph and Mildred Sonshine Family Centre for Head and Neck Diseases, Department of Otolaryngology-Head and Neck Surgery Program, Mount Sinai Hospital, Joseph & Wolf Lebovic Health Complex, 600 University Avenue, Room 413, Toronto, ON, M5G 1X5, Canada

**Keywords:** ESLI, EpCAM, Ep-ICD, EpEx, Papillary thyroid Microcarcinoma, Aggressiveness, Metastatic

## Abstract

**Background:**

Thyroid cancer is among the fastest growing malignancies; almost fifty-percent of these rapidly increasing incidence tumors are less than or equal to 1cm in size, termed papillary thyroid microcarcinoma (PTMC). The management of PTMC remains a controversy due to differing natural history of these patients. Epithelial cell adhesion molecule (EpCAM) is comprised of an extracellular domain (EpEx), a single transmembrane domain and an intracellular domain (Ep-ICD). Our group reported nuclear Ep-ICD correlated with poor prognosis in thyroid cancer (Ralhan et al., BMC Cancer 2010,10:331). Here in, we hypothesized nuclear and cytoplasmic accumulation of Ep-ICD and loss of membranous EpEx may aid in distinguishing metastatic from non-metastatic PTMC, which is an important current clinical challenge. To test our hypothesis, Ep-ICD and EpEx expression levels were analyzed in PTMC and the staining was correlated with metastatic potential of these carcinomas.

**Methods:**

Thirty-six PTMC patients (tumor size 0.5 - 1cm; metastatic 8 cases and non-metastatic 28 cases) who underwent total thyroidectomy were selected. The metastatic group consisted of patients who developed lymph node or distant metastasis at diagnosis or during follow up. The patients’ tissues were stained for Ep-ICD and EpEx using domain specific antibodies by immunohistochemistry and evaluated.

**Results:**

PTMC patients with metastasis had higher scores for nuclear and cytoplasmic Ep-ICD immunostaining than the patients without metastasis (1.96 ± 0.86 vs. 1.22 ± 0.45; p = 0.007 and 5.37 ± 0.33 vs. 4.72 ± 1.07; p = 0.016, respectively). Concomitantly, the former had lower scores for membrane EpEx than the non-metastatic group (4.64 ± 1.08 vs. 5.64 ± 1.51; p = 0.026). An index of aggressiveness, Ep-ICD subcellular localization index (ESLI), was defined as sum of the IHC scores for accumulation of nuclear and cytoplasmic Ep-ICD and loss of membranous EpEx; ESLI = [Ep − ICD_nuc_ + Ep − ICD_cyt_ + loss of membranous EpEx]. Notably, ESLI correlated significantly with lymph node metastasis in PTMC (p = 0.008).

**Conclusion:**

Nuclear and cytoplasmic Ep-ICD expression and loss of membranous EpEx were found to correlate positively with metastasis in PTMC patients. In addition, ESLI had the potential to identify metastatic behavior in PTMC which could serve as a valuable tool for solving a current dilemma in clinical practice.

## Background

Thyroid cancer represents about 1% of all new malignant diseases and is the most common endocrine malignancy [1]. Ninety-four percent of thyroid cancers are differentiated carcinomas, mainly papillary thyroid cancer (PTC) [1,2]. In the United States, the incidence of thyroid cancer was approximately 37 200 new cases per year in 2009 [3] and the estimated number of cases for the year 2012 is 56 460 (National Cancer Institute 2012). According to SEER 2012, thyroid cancer is among the fastest growing malignancies with an increasing significant trend of 6.6 (where significance indicates that there is 95% confidence that the increase is real over the period of time measured and not due to chance alone) ( http://seer.cancer.gov). The sharp elevation within the past decade can be attributed, in part, to the more frequent use of high-resolution ultrasound guided FNA with the advantage of better accuracy and accessibility. Forty-nine percent of growing incidence of thyroid cancer has been credited to tumors with a size of 1cm or smaller [4]. According to the World Health Organization classification, papillary thyroid microcarcinoma (PTMC) is defined as papillary thyroid cancer of size less than or equal to 1 cm in maximal diameter [5]. The prevalence of PTMC ranges from 3.5-35.6%, and its incidence has demonstrated an upward trend in all age groups [3,6,7].

PTMCs can be classified into two broad clinical categories. The majority of PTMCs fall in the non-aggressive group which do not cause any symptoms throughout a patient’s life and are essentially very low risk thyroid carcinomas. However, there have been reports of patients presenting with cervical lymph node metastasis of thyroid origin without a palpable thyroid nodule [8] or presenting with concomitant cervical lymph node and distant metastasis [9,10]. The survival rate of PTMC is excellent; cancer related deaths are only 0.34% [11]. However, 2.4% – 20% of PTMCs have locoregional recurrence [11,12]. Management of PTMC is still a topic of hot debate due to varying natural history of PTMC. The conservative “wait and watch” treatment for PTMC has been advocated due to its benign clinical course [13]. On the contrary, surgery has been recommended as the treatment of choice for PTMC [14-16]. A variety of clinical and pathological criteria are used to determine the aggressive potential as well as risk of recurrence in PTMC such as age, sex, focality, and lymph node metastasis at diagnosis. However, PTMC is frequently an incidental finding and the availability of these clinicopathological criteria is circumspect at the time. Haymart et. al observed that 78.5 percent of patients had PTMC as an incidental finding on postsurgical pathology report [17]. In addition, the use of ultrasonography to assess the above-mentioned criteria is restricted by its own limitations of being operator dependent and not accurate or sensitive enough; the sensitivity of ultrasonographic diagnosis for multifocality and lymph node metastasis in the lateral compartment are 52.9% and 38.3%, respectively [18]. Thus, it is important to establish a definite marker which would either complement the existing criteria or act alone to differentiate aggressive PTMC from non-aggressive cases and serve as an invaluable tool in clinical practice. Single-center retrospective study of a cohort of 1669 patients with PTMC managed from 1960 to 2007 proposed a scoring system to classify recurrence risk [19]. The recurrence probability of pT3 PTMC appeared lower if radioiodine ablation was performed, while in PTMC_Nx_ (lymph node status not known) patients, multifocality was important in planning therapeutic strategies [19].

At present, there is a dearth of validated biomarkers that have crossed the bridge from laboratory to clinic. BRAF mutation has shown promising results in predicting prognosis in conventional PTC, however its prevalence is distinctly lower (18%) in PTMC smaller than 5mm in diameter [20]. Cyclin D1 nuclear expression also had inconclusive results [21,22]. S100A4 expression significantly correlated with extra thyroidal extension and multifocality in PTMC, but despite extensive studies, this protein has not translated into a reliable biomarker in the clinics [22]. Oligonucleotide array analysis revealed that cell adhesion molecules were consistently up-regulated in PTMC [23]. Further, another significant finding was the absence of differences in the gene expression profiles of PTMC and PTC, hinting at the possibility that some PTMC might represent an early detected stage of conventional PTC as opposed to being a distinct entity [24]. This presents the plausibility that a biomarker which has given reliable results in predicting prognosis in PTC might be extrapolated for the same use in PTMC.

Epithelial cell adhesion molecule (EpCAM) is a 40 kDa transmembrane glycoprotein, comprised of an extracellular domain (EpEx), a single transmembrane domain and a short 26 amino acid intracellular domain (Ep-ICD) [25]. Ep-ICD has been demonstrated to be frequently overexpressed in human malignancies by our group [26]. EpCAM plays a major role in a multitude of processes including cell adhesion, proliferation, differentiation, cell cycle regulation and is implicated in cancer signaling. Recently, we reported nuclear and cytoplasmic accumulation of Ep-ICD and loss of membranous EpEx to be a marker for poor prognosis in thyroid cancer [27]. Taking all of the above into consideration, we sought to explore the application of EpCAM in answering the question of aggressive potential in PTMC. The aim of this study was to discern Ep-ICD and EpEx expression in metastatic and non-metastatic PTMC. In addition, we defined a composite representation of EpCAM staining, ESLI (Ep-ICD subcellular localization index), as the sum of loss of membranous EpEx staining and nuclear and cytoplasmic Ep-ICD accumulation. ESLI has recently been validated by us in a cohort of 200 patients as a reliable tool for identifying aggressive behavior in PTC [28]. In view of the above stated similar gene expressions of PTC and PTMC, we sought to investigate the ability of this marker to better answer the clinical question at hand.

## Methods

### Patients and materials

This study was approved by the Research Ethics Board of Mount Sinai Hospital. The histopathology reports of patients who underwent thyroid surgery at Mount Sinai Hospital were reviewed. Only patients who had total thyroidectomy as their primary mode of treatment were selected in order to accurately assess the focality of PTMC. Further inclusion criteria consisted of PTMC size more than or equal to 5 mm which was based on literature survey that demonstrated more aggressive behavior in PTMC of size ≥ 5 mm [22,29]. Cases with thyroid surgery other than total thyroidectomy or tumor size smaller than 5mm were excluded. Based on these criteria, 36 PTMC patients were identified between 2006 and 2011. All thirty-six slides were reviewed by the pathologist (CM) to confirm the diagnosis of PTMC. IHC for Ep-ICD and EpEx was performed in all these tissue sections as previously described by us [27]. During the follow-up period, 2 of these patients had persistent disease (no remission), 1 had recurrent disease (relapse after remission) and the remaining 33 were disease free during the defined time interval of 5 years.

### Antibodies

Anti-human-EpCAM mouse monoclonal antibody MOC-31 (AbD Serotec, Oxford, UK) recognizes an extracellular component (EpEx) in the amino-terminal region of EpCAM. α-Ep-ICD antibody 1144 [Epitomics Inc. (Burlingame, CA)] recognizes the intracellular domain of EpCAM, Ep-ICD.

### Immunohistochemistry for EpEx and Ep-ICD expression in PTMCs

Serial PTMC tissue sections (4 μm thickness) were deparaffinized, hydrated in xylene and graded alcohol series. Antigen retrieval was carried out using a microwave oven in 0.01 M citrate buffer, pH 6.0; thereafter the slides were treated with 0.3% H_2_O_2_ at room temperature for 30 minutes to block the endogenous peroxidase activity. After blocking for non-specific binding with horse or goat serum, the sections were incubated with anti-human antibodies -EpEx mouse monoclonal antibody MOC-31 (dilution 1:200), or α- Ep-ICD rabbit monoclonal antibody 1144 (dilution 1:200) respectively and biotinylated secondary antibody (horse antimouse or goat anti-rabbit respectively) for 30 minutes. The sections were subsequently incubated with VECTASTAIN Elite ABC Reagent (Vector laboratories, Burlington, Ontario, Canada) and diaminobenzidine was used as the chromogen. Hematoxylin was used as the counterstain for nuclei. The primary antibody was replaced with isotype specific IgG in PTMC used as the negative control. Colon cancer tissue sections known to express Ep-ICD or EpEx were used as positive controls in each batch of IHC analysis.

### Evaluation of immunohistochemical staining

Sections were scored as positive if epithelial cells showed immunopositivity in the plasma membrane, cytoplasm, and/or nucleus when observed by two independent evaluators who were blinded to the clinical outcome. These sections were scored as follows: 0, < 10% cells; 1, 10–30% cells; 2, 31–50% cells; 3, 51–70% cells; and 4, > 70% cells showed immunoreactivity. Sections were also scored semi-quantitatively on the basis of intensity as follows: 0, none; 1, mild; 2, moderate; and 3, intense. Finally, a total score (ranging from 0 to 7) was obtained by adding the scores of percentage positivity and intensity for the thyroid cancer [26,27]. Loss of membranous EpEx was calculated as the maximum total score of 7- score for membrane EpEx.

### Ep-ICD subcellular Localization Index (ESLI)

ESLI was defined as sum of the IHC scores for accumulation of nuclear and cytoplasmic Ep-ICD and loss of membranous EpEx; ESLI = [Ep − ICD_nuc_ + Ep − ICD_cyt_ + loss of membranous EpEx] [28].

### Statistical analysis

Statistical analysis was performed with SPSS software version 20.0. Categorical variables were presented by number of cases and percentage. Fisher’s exact test was used when comparing frequencies between groups. Continuous variables were presented by mean ± standard deviation (SD) or median with range. Independent T test was used when comparing continuous variables between groups. Probability values less than 0.05 were considered statistically significant.

## Results

### Patient follow-up

Thirty-six patients met the inclusion and exclusion criteria of the study. Eight patients were classified in the metastatic group. All patients in the metastatic group had lymph node metastasis at diagnosis. Two patients had persistent disease and one patient had recurrence. No patient had distant metastasis or death during the follow up period. There were 28 patients in the non-metastatic group.

### Clinicopathological features of metastatic PTMC and non-metastatic PTMC

Clinicopathological features were compared between metastatic and non-metastatic groups. Patients with metastasis had advanced TNM stage compared to those without (p = 0.03) and I-131 treatment was administered more in the metastatic group (87.5% vs. 14.3%; p < 0.001) (Table 1). Patients in the metastatic group were younger in age (41.8 ± 12.5 vs. 55.8 ± 10.7 years; p = 0.003; Table 2). No significant differences were found between the two groups in terms of other clinicopathological variables, including patients’ gender, tumor size, histological subtype, multifocality, extrathyroidal extension and duration of follow up (Table 1 and Table 2).

**Table 1:** Patient characteristics distribution of the metastatic and non-metastatic PTMC

**Patient characteristics**	**Metastatic (n = 8)**	**Non-metastatic (n = 28)**	**P-value**
Gender			
Female	4 (50%)	23 (82.1%)	0.086
Male	4 (50%)	5 (17.9%)	
Histological subtype			
Classical	5 (62.5%)	11 (39.3%)	0.742
Follicular	3 (37.5%)	14 (50%)	
Oncocytic	0 (0)	2 (7.1%)	
Diffuse sclerosing	0 (0)	1 (3.6%)	
Multifocal	6 (75%)	16 (57.1%)	0.441
Lymph node metastasis at diagnosis	8 (100%)	0	<0.001
Extrathyroidal extension	2 (25%)	3 (10.7%)	0.305
Angioinvasion	3 (37.5%)	2 (7.1%)	0.061
TNM stage			
I&II	4 (50%)	26 (92.9%)	0.03
III&IV	4 (50%)	2 (7.1%)	
I-131 No.			
0	1 (12.5%)	24 (85.7%)	<0.001
≥ 1	7 (87.5%)	4 (14.3%)	

**Table 2:** Patient characteristics distribution (Mean ± SD) of the metastatic and non-metastatic group

**Patient characteristics**	**Metastatic (n = 8)**	**Non-metastatic (n = 28)**	**P-value**
Age (year)	41.8 ± 12.5	55.8 ± 10.7	0.003
Tumor size (cm)	0.86 ± 0.14	0.76 ± 0.15	0.111
Duration of follow up (months)	31 ± 19	25 ± 19	0.238

### Immunohistochemical Ep-ICD and EpEx expression in metastatic PTMC and non-metastatic PTMC

**Figure 1:**
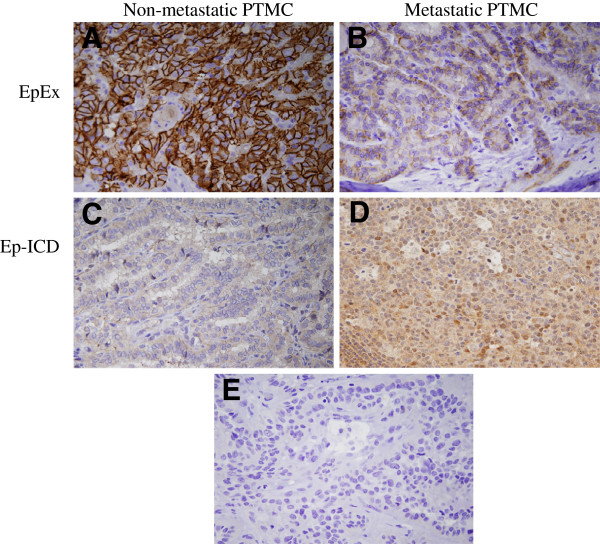
**Immunohistochemical analysis of EpEx and Ep-ICD expression in papillary thyroid microcarcinoma.** The representative photomicrographs show immunostaining of EpEx and Ep-ICD in PTMC. Strong membranous EpEx immunostaining was observed in the non-metastatic group (**A**), whereas decreased staining of membranous EpEx was observed in aggressive PTMC cases (**B**). The non-metastatic PTMC show predominant cytoplasmic localization of Ep-ICD and no detectable nuclear Ep-ICD staining (**C**), while the aggressive cases show nuclear and strong cytoplasmic Ep-ICD accumulation (**D**). (**E**) depicts the negative control. Original magnification x 400.

Patients with metastasis had reduced levels of membrane EpEx than those without metastasis (4.64 ± 1.08 vs. 5.64 ± 1.51; p = 0.026) (Table 3 and Figure 1A and B). Patients with metastatic PTMC had higher scores of Ep-ICD nucleus and cytoplasm than the non-metastatic group (1.96 ± 0.86 vs. 1.22 ± 0.45; p = 0.007 and 5.37 ± 0.33 vs. 4.72 ± 1.07; p = 0.016, respectively; Table 3 and Figure 1C and D). PTMC with lymph node metastasis showed higher ESLI scores as compared to the non-metastatic group (9.69 ± 2.01 vs. 7.30 ± 2.39 respectively; p = 0.008; Table 3).

**Table 3:** Subcellular localization of Ep-ICD and EpEx in metastatic and non-metastatic PTMC

**Protein localization**	**Metastatic (mean ± SD)**	**Non-metastatic (mean ± SD)**	**P-value**	**AUC**	**A.sig**
EpEx cytoplasm	3.00 ± 1.67	1.91 ± 1.16	0.095	0.694	0.098
EpEx membrane	4.64 ± 1.08	5.64 ± 1.51	0.026	0.243	0.029
Loss of membranous EpEx	2.36 ± 1.08	1.36 ± 1.51	0.026	0.757	0.029
Ep-ICD nucleus	1.96 ± 0.86	1.22 ± 0.45	0.007	0.783	0.016
Ep-ICD cytoplasm	5.37 ± 0.33	4.72 ± 1.07	0.016	0.766	0.024
Ep-ICD membrane	1.30 ± 1.07	1.93 ± 1.61	0.348	0.391	0.351
ESLI (loss of membranous EpEx + Ep-ICDnuc + Ep-ICD cyto)	9.69 ± 2.01	7.30 ± 2.39	0.008	0.808	0.009

### ESLI is a potential marker for aggressive PTMC

**Figure 2:**
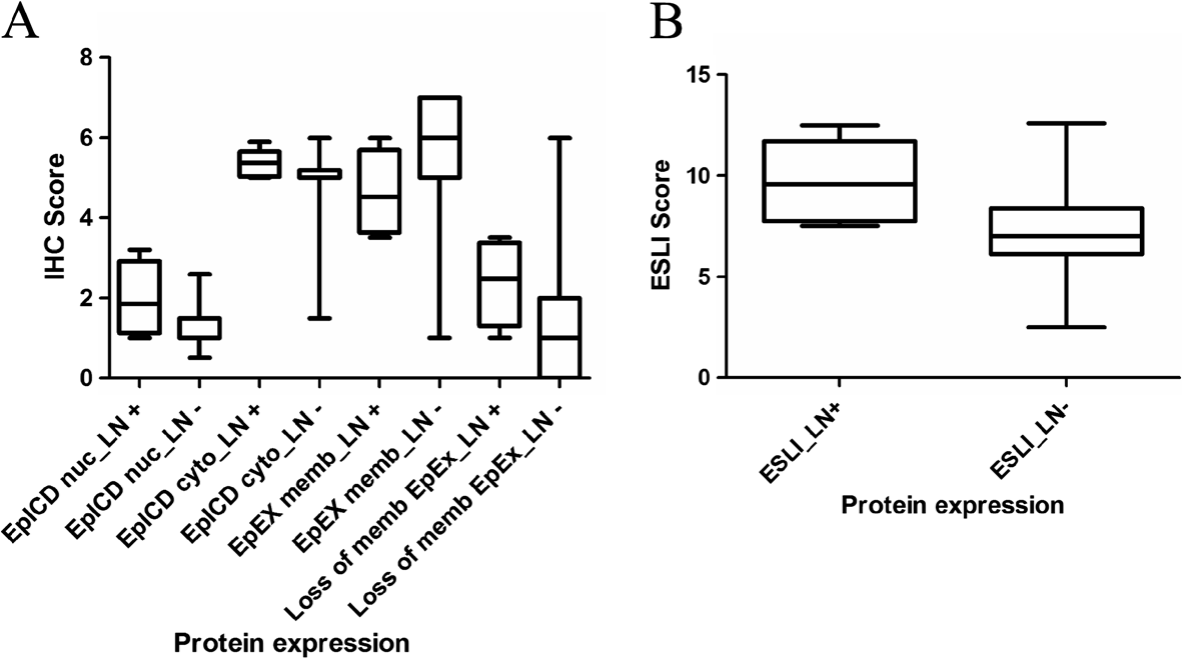
**Box plot analysis of Ep-ICD and EpEx staining in PTMC.** Comparison of Ep-ICD and EpEx immunostaining in metastatic and non-metastatic PTMCs shows increase in nuclear and cytoplasmic Ep-ICD and loss of membranous EpEx in the metastatic PTMC. EpEx membrane is correspondingly reduced in the metastatic PTMC group (**A**). ESLI showed significant correlation with lymph node metastasis (**B**). Abbreviations in the figure: LN+, lymph node metastasis; LN-, no lymph node metastasis; nuc, nucleus; cyto, cytoplasm; memb, membrane.

**Figure 3:**
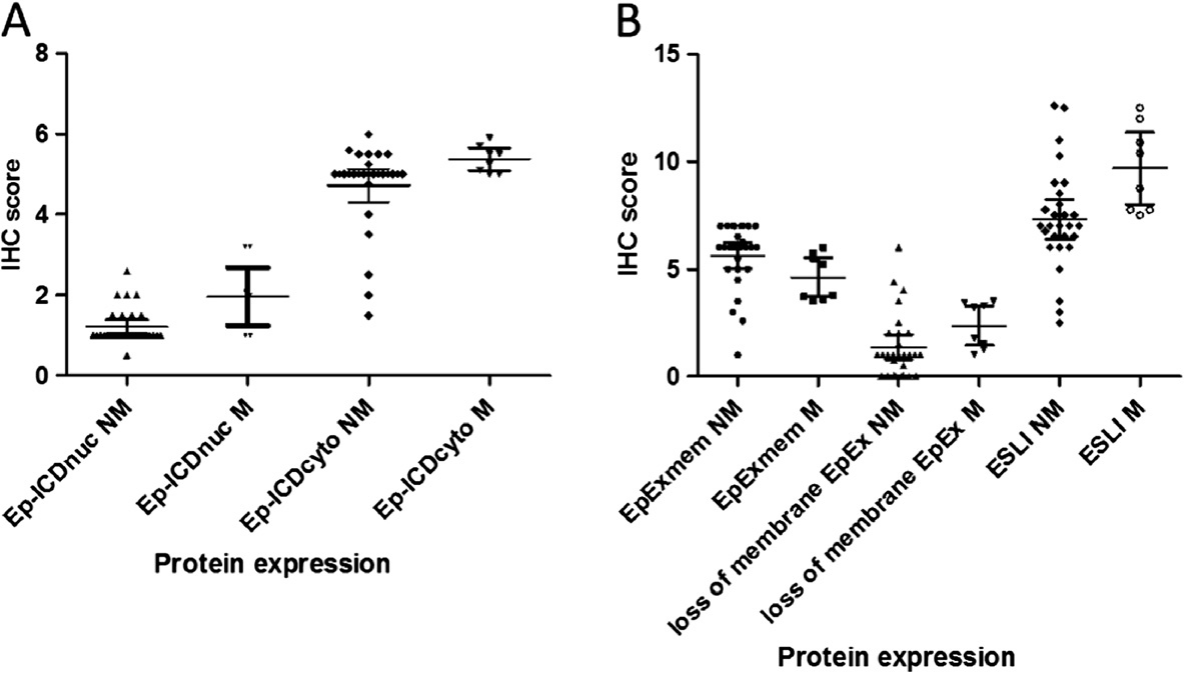
**Dot plot analysis of Ep-ICD and membranous EpEx expression in papillary thyroid microcarcinoma patients.** Dot plot shows the distribution of total IHC scores for Ep-ICD (**A**); EpEx and ESLI (**B**). The vertical axis gives the IHC score as described in the Methods section. Increased nuclear expression of Ep-ICD was more frequently observed in the metastatic PTMC group as compared to the non-metastatic PTMCs (**A**). High membrane EpEx expression was observed in non-metastatic PTMCs, whereas decreased membranous expression of EpEx was observed in most of the aggressive PTMC cases analyzed (**B**). An increase in ESLI was observed in the metastatic PTMC group as compared to the non-metastatic PTMCs (**B**). Abbreviations: NM, non-metastatic; M, metastatic.

**Figure 4:**
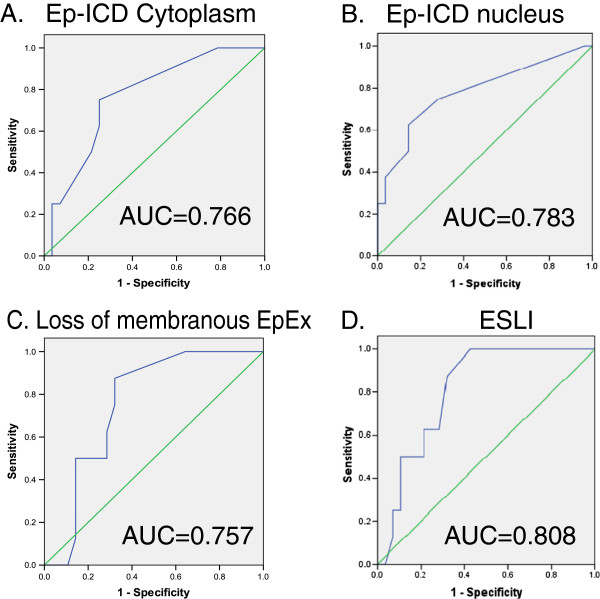
**ROC curve analysis of cytoplasmic and nuclear Ep-ICD, loss of membranous EpEx and ESLI in PTMC.** The vertical axis represents sensitivity and the horizontal axis represents 1-specificity in ROC curves of cytoplasmic (**A**) and nuclear Ep-ICD (**B**), loss of membranous EpEx (**C**) and ESLI (**D**). The area under the curve (AUC) values and significance values for the two groups are summarized in Table 3.

Box plot analysis revealed an increasing trend of Ep-ICD cytoplasm, Ep-ICD nucleus, loss of membranous EpEx as well as ESLI with lymph node metastasis (Figure 2A and B) which adds credence to our hypothesis that aggressive behavior in PTMC is characterized by loss of surface EpCAM and accumulation of its intracellular domain Ep-ICD. Dot plot analysis revealed similar trend of accumulation of Ep-ICD in nucleus and cytoplasm in metastatic PTMC (Figure 3A). Non-metastatic PTMC had strong membrane EpEx staining which was reduced or lost with more aggressive characteristics (Figure 3B). ROC curve analysis showed area under the curve (AUC) for Ep-ICD cytoplasm, Ep-ICD nucleus and loss of membranous EpEx were 0.766 (p = 0.024), 0.783 (p = 0.016) and 0.757 (p = 0.029) respectively (Figure 4A, B and C respectively; Table 3). ESLI, an index of aggressiveness, showed an AUC of 0.808 and was associated with lymph node metastasis (p = 0.009) (Table 3 and Figure 4D).

## Discussion

PTMC patients have excellent survival statistics and a very low mortality rate of 0.34%, therefore, the current therapeutic strategies are mainly focused on reducing morbidity such as tumor recurrence or metastasis. PTMC patients with cervical lymph node metastasis at diagnosis had more events of recurrence than PTMC patients without nodal metastasis [10,14]. Moreover, the presence of lymph node metastasis at diagnosis increased the relative risk of distant metastasis 11.2 fold [10]. Thus, nodal metastasis at presentation is a prognostic marker for tumor recurrence and distant metastasis in PTMC. However, lymph node sampling is not routinely performed in all thyroid surgeries, especially in cases where thyroid cancer is not suspected prior to surgery, which compounds the issue in the finding of incidental PTMC.

Current American Joint Committee on Cancer (AJCC) TNM staging recommends using a select few clinicopathological variables to determine prognosis in thyroid cancer patients [30]. Age of the patient is a well-known prognostic factor in differentiated thyroid cancer with age more than or equal to 45 years having a worse outcome [30]. Notably, PTMC patients with lymph node metastasis were younger than PTMC patients without lymph node metastasis in this study. This finding has also been confirmed in a recent study by Zhang et al. [31] that supports our observations suggesting younger patients may have more aggressive disease, especially in case of occult PTMC. These challenges along with presence of divisive data on the prognosis of PTMC urge accurate categorization of the aggressive subset of this malignancy. Hence, there is an urgent unmet need to identify a universal undisputed biomarker that, independently or in conjunction with known criteria, serves to stratify PTMCs according to their metastatic potential.

It is imperative to understand the mechanism of EpCAM activation and the relationship between alterations in the levels of its intra- and extra- cellular domains. The shedding of extracellular EpEx triggers intramembranous cleavage of the remaining EpCAM molecule by tumor necrosis factor-alpha convertase (TACE) and γ-secretase complex (containing presenilin2 as a catalytic subunit). This is turn activates the release of the intracellular domain Ep-ICD which binds to β-catenin and FHL2 to translocate into the nucleus. The ensuing complex initiates a cascade of events leading up to and participating in increased gene transcription and cell proliferation [32].

In this study, metastatic PTMCs showed higher expression of nuclear Ep-ICD compared to the non-metastatic PTMCs. Moreover, membranous EpEx was reduced in the PTMC group with lymph node metastasis. Notably, in support of our findings, loss of membranous EpCAM has been significantly associated with the presence of lymph node metastasis in colorectal cancer as well [33]. Furthermore, we introduced a new index of aggressiveness, ESLI, in PTMC that demonstrated effectiveness of the combination of subcellular (cytoplasmic and nuclear) localized staining in distinguishing the metastatic PTMC from non-metastatic, as compared to Ep-ICD and EpEx alone. The findings of our study need to be validated in a larger cohort to be applicable in clinical practice. A larger number of cases could also help correlating Ep-ICD expression with survival in PTMC patients, which was a limitation of this study. The risk of lymph node recurrence increased when multifocality was present [10], which could not be established in this study due to limited sample size.

A randomized control trial would be the best way to evaluate the relevance of this marker in the clinics. The novelty of our study lies in the fact that it is the first to explore the expression of Ep-ICD, EpEx and ESLI in PTMC. In addition, we bring a composite marker in the form of ESLI which has proven more valuable than Ep-ICD or EpEx alone, in answering the pertinent question of aggressive potential of PTMC. The use of ESLI for predicting lymph node metastasis in PTMC could prevent future recurrences or distant metastasis by allowing for more aggressive treatment. The ability to differentiate indolent PTMC from metastatic would help conserve vital time and resources by effectively directing aggressive management to the patients who require it while at the same time, saving patients with essentially benign disease from unnecessary treatment. Thus, Ep-ICD, EpEx and ESLI are plausible candidate markers to elucidate the myriad unrequited queries that surround the PTMC enigma.

## Conclusion

This study provides new evidence in support of the potential of Ep-ICD and EpEx when incorporated with ESLI to serve as markers for identification of aggressive PTMC from non-aggressive PTMC.

## Abbreviations

EpCAM: Epithelial cell adhesion molecule; EpEx: Extracellular domain; Ep-ICD: Intracellular domain of EpCAM; PTC: Papillary thyroid cancer; PTMC: Papillary thyroid microcarcinoma; IHC: Immunohistochemistry; ESLI: Ep-ICD subcellular localization index; AUC: Area under the curve; ROC: Receiver-operating characteristic.

## Competing interests

The authors declare that they have no competing interests.

## Authors’ contributions

TK and RR conceptualized and designed the study. TK and IK conducted the study, carried out the experimental work, data analysis, photomicrography and wrote the manuscript. TK and IK conducted the chart reviews and provided clinical data for all the patients. CM performed all the histopathological evaluation embodied in this study. PGW conceptualized the study, provided the infrastructure and funding support, supervised the work and data analysis and edited the manuscript. All the authors read and approved the manuscript.

## Authors’ information

Ranju Ralhan and Paul G. Walfish are senior authors in this study.

## Pre-publication history

The pre-publication history for this paper can be accessed here:

http://www.biomedcentral.com/1471-2407/12/523/prepub
